# A Rare Case of Bilateral Maxillary Supplemental Lateral Incisors

**DOI:** 10.7759/cureus.93769

**Published:** 2025-10-03

**Authors:** Divya Singla, Yenika Manchanda, Mitasha Sachdeva, Harvinder Bhangu, Vinay Dua

**Affiliations:** 1 Department of Orthodontics, National Dental College & Hospital, Mohali, IND

**Keywords:** dental anomaly, hyperdontia, non-syndromic supernumerary teeth, supplemental teeth, tooth duplication

## Abstract

Supplemental teeth are uncommon and often go undetected during routine dental examinations, especially when they resemble normal teeth in shape and size. In this case, a 17-year-old male presented with a rare finding: bilateral supplemental maxillary lateral incisors. Although he reported no discomfort, the presence of these extra teeth had the potential to affect his bite. On examination, the additional lateral incisors looked almost identical to the natural ones and showed complete root development on radiographs. After a thorough evaluation, the treatment plan involved extracting the supplemental teeth and initiating fixed orthodontic therapy to manage spacing and tooth alignment. This case highlights the need for careful diagnosis and personalized treatment planning, even when anomalies appear harmless or go unnoticed.

## Introduction

Supernumerary teeth can appear on one or both sides of the dental arches, with the premaxillary region being the most frequently affected site [1]. The precise cause of hyperdontia is still not clearly understood. Several theories have been proposed to explain its origin, including the dichotomy theory, the atavism hypothesis, and the most widely accepted dental lamina hyperactivity theory [2].

The dichotomy theory suggests that a tooth bud splits (dichotomizes) into two equal or unequal parts; this results in either twin teeth (if equal division) or one normal and one rudimentary/smaller supernumerary tooth (if unequal) [2].

The Atavism hypothesis is based on the concept of phylogenetic reversion (reappearance of ancestral traits), suggesting that supernumerary teeth represent a throwback to our ancestors, who had a larger number of teeth (e.g., primitive humans had more incisors and premolars). Thus, extra teeth are considered a regression to evolutionary dentition patterns [2].

Dental lamina hyperactivity theory proposes that remnants of the dental lamina (responsible for tooth development) show localized hyperactivity, giving rise to additional tooth buds. These buds develop into supernumerary teeth, which can vary in shape (conical, tuberculate, and supplemental) [2,3].

The prevalence of extra teeth in the primary dentition ranges from 0.3% to 0.6%, which is about five times lower than in the permanent dentition [4]. This reduced occurrence in primary teeth may be due to unnoticed exfoliation or early extraction [4]. The maxilla is significantly more likely to develop supernumerary teeth (by a factor of eight) compared with the mandible [5]. Among all supernumerary teeth, the occurrence of permanent lateral incisors as supernumeraries is relatively rare, with mesiodens (present between central incisors) being most common, followed by paramolars (fourth molars) and mandibular premolars [6].

Studies indicate that the prevalence of supernumerary teeth in the permanent dentition varies between 0.5% and 5.3% [7]. Male patients tend to have a higher incidence, with a male-to-female ratio of approximately 2:1, although no significant gender difference is observed in primary dentition [7,8].

A classification of numeric dental anomalies was published by Tomes [9], who defined the following: (1) Supplemental: Tooth characterized by the same form and function of adjacent teeth with no anatomical differences. (2) Supernumerary: Tooth characterized by an atypical anatomic form; often these teeth are smaller than normal.

Supernumerary teeth can be classified into four main morphological types: conical, tuberculate, supplemental, and odontomes [3]. Supplemental teeth are those that resemble normal teeth in both shape and size and usually appear at the end of a tooth series. The most commonly found supplemental tooth is the maxillary permanent lateral incisor [10]. These teeth also represent the most frequent type of extra teeth found in the primary dentition [10].

In the case discussed here, bilateral supplemental lateral incisors were found in the upper jaw. Supplemental lateral incisors may erupt or remain unerupted and can occur either unilaterally or bilaterally, in both primary and permanent dentitions. Their presence can negatively affect the appearance of the anterior teeth and may lead to various occlusal problems [11]. These include increased overjet, dental crowding or ectopic eruptions, midline deviations, and psychological effects such as reduced self-confidence [11].

Whether erupted or impacted, additional teeth can cause a range of clinical issues. These may include prolonged retention of primary teeth, delayed or impacted eruption of permanent teeth, abnormal tooth positioning or rotation, and pathological conditions such as odontogenic cyst formation (e.g., dentigerous or follicular cysts) [12]. Other complications may involve abnormal spacing (diastema) or, in rare cases, resorption of the roots of adjacent permanent teeth. Among these, root resorption is one of the most serious and common consequences if the condition remains untreated [12].

## Case presentation

A 17-year-old male patient visited the dental hospital in Derabassi for a routine dental check-up. The patient presented with a complete set of permanent teeth in both the maxillary and mandibular arches, along with bilateral supplemental lateral incisors that closely resembled the natural maxillary lateral incisors in form. The patient was unaware of the presence of any additional teeth. Clinical examination revealed no significant findings in the patient’s family or medical history.

Spacing was noted in both the upper and lower anterior regions. The fremitus test was positive in relation to bilateral supplemental lateral incisors. The crown and root morphology of the maxillary right lateral incisor was identical to its supplemental counterpart. The mesiodistal width and crown length of the right lateral incisor were equal to those of the supplemental tooth. In contrast, the left lateral incisor and its supplemental tooth showed differences in crown width and length.

Flossing between teeth 12 and 12S allowed easy passage, indicating a clear separation and confirming the presence of two distinct teeth. Radiographic evaluation revealed normal crown anatomy, including enamel, dentin, pulp, and root structures, with a healthy periodontium associated with both the right lateral incisor and its supplemental counterpart. While both teeth displayed similar morphology, the supplemental tooth on the left side showed signs of crown attrition and root resorption. These anomalies contributed to several aesthetic and functional concerns, including increased incisor proclination, excessive incisor display, a non-optimal lip line, a non-consonant smile arc, and potentially incompetent lips.

**Figure 1:**
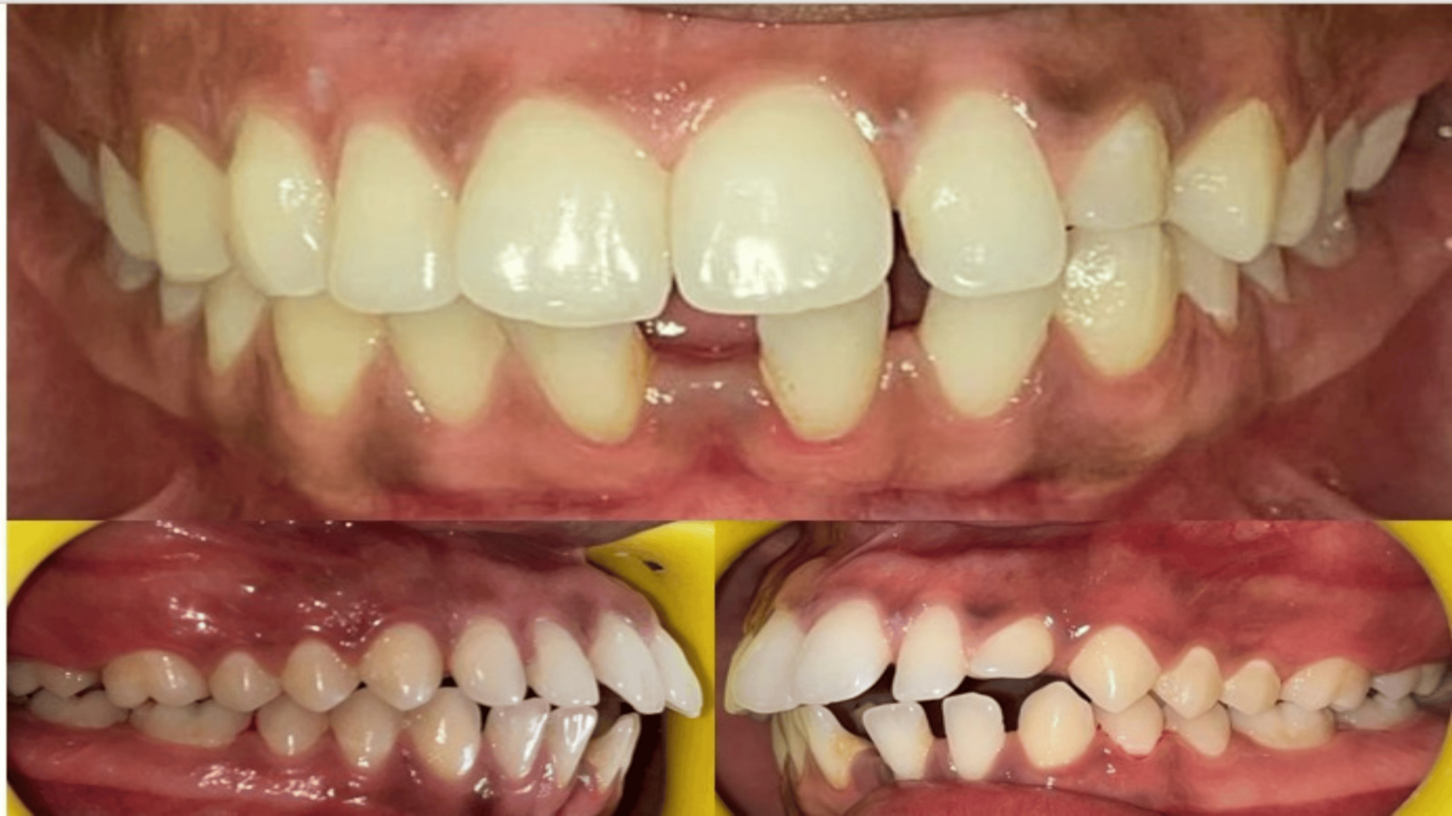
Intraoral photographs

**Figure 2:**
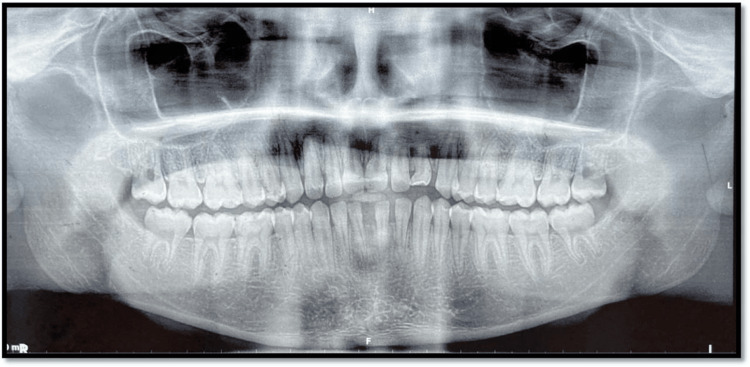
Orthopantomogram

**Figure 3:**
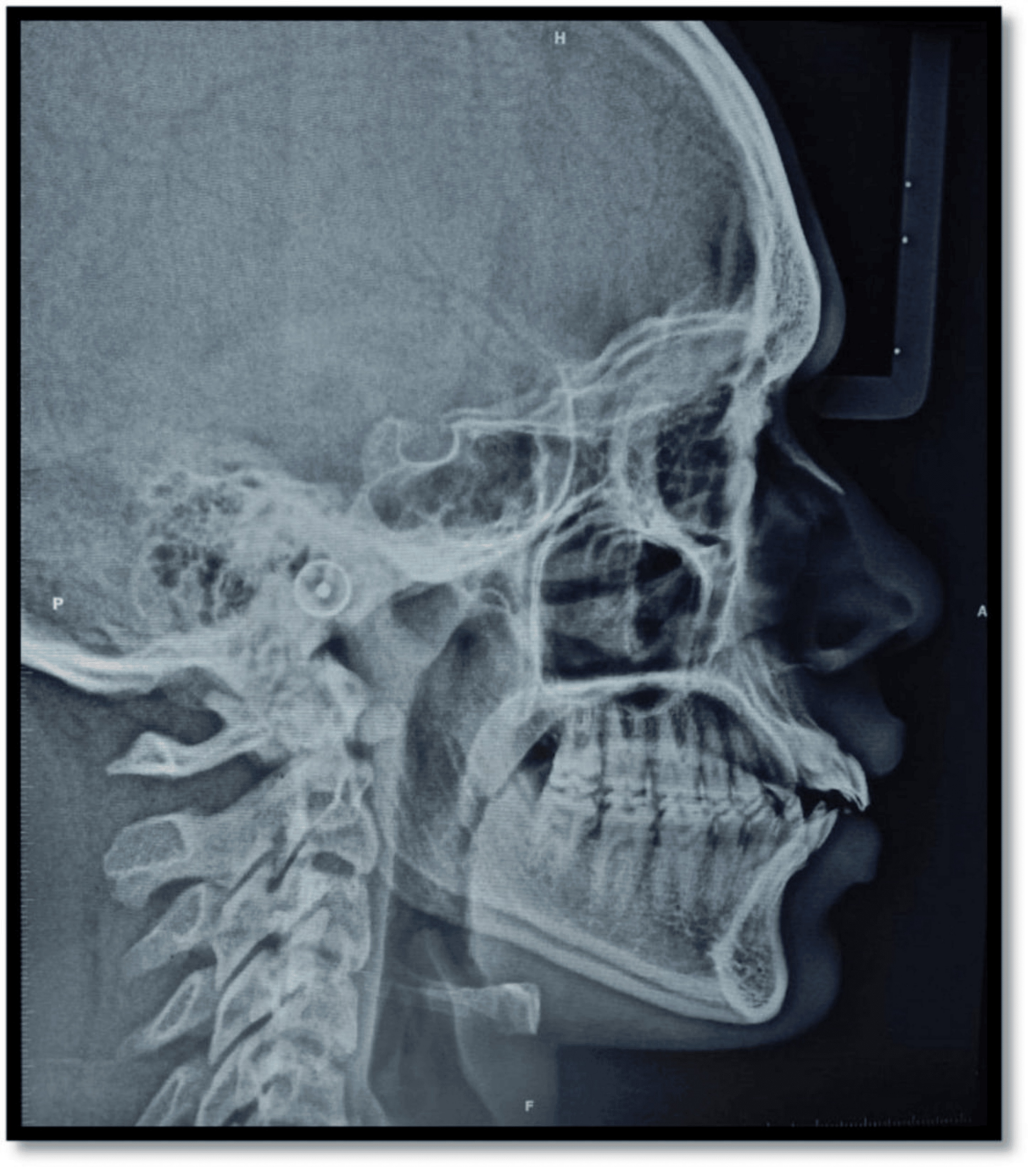
Lateral cephalogram

Additional diagnostic tools included clinical photographs (Figure 1), orthopantomogram (OPG) (Figure 2), and a lateral cephalogram (Figure 3). Cast analysis also revealed generalized spacing in both arches, proclined upper and lower incisors, increased overjet, and pit and fissure caries in teeth 36, 37, 46, and 47. The patient had a convex facial profile, an acute nasolabial angle, and potentially competent lips.

The recommended treatment plan involved the extraction of the supplemental maxillary lateral incisor followed by space closure using fixed mechanotherapy. As the orthodontic treatment is still in progress, final outcomes could not be presented. Detailed results will be reported in a subsequent article.

## Discussion

Supernumerary teeth represent an important developmental anomaly, with a reported prevalence of 0.5%-5.3% in the permanent dentition and a male predominance. Among these, supplemental teeth that resemble normal morphology are distinctly uncommon. The maxillary lateral incisor is the most frequent site for supplemental teeth in the permanent dentition, yet bilateral occurrences remain rare, comprising less than 10% of all reported cases. Our patient thus represents an unusual presentation of bilateral supplemental maxillary lateral incisors, with both teeth fully erupted and morphologically similar to their natural counterparts [13].

**Figure 4:**
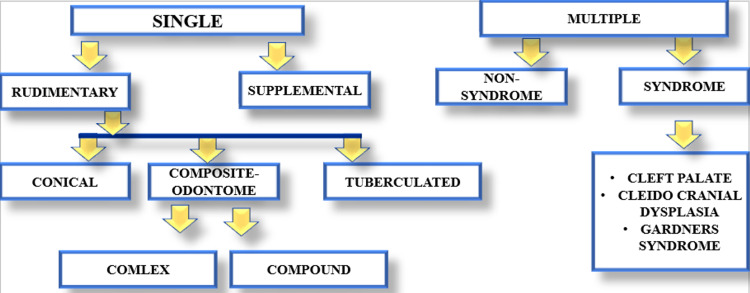
Classification of supernumeraries

The classification of supernumeraries (Figure 4) by a few authors is as follows [14]:

Embryological and etiological considerations

Several hypotheses attempt to explain the formation of supernumerary teeth. The dichotomy theory suggests splitting of the tooth bud, while the atavism hypothesis attributes it to evolutionary reversion. However, the most widely accepted explanation is localized hyperactivity of the dental lamina, leading to additional tooth buds and the development of morphologically normal supplemental teeth. Recent genetic studies also highlight familial aggregation and associations with mutations affecting the Wnt/β-catenin signaling pathway, supporting a multifactorial origin [14,15].

Syndromic associations

Supernumerary teeth frequently occur in association with syndromes such as cleidocranial dysplasia and Gardner syndrome and certain craniofacial clefting disorders. A thorough syndromic screening was conducted in our patient: no dysmorphic skeletal features, cutaneous lesions, or family history suggestive of syndromic involvement were observed. The medical and family histories were unremarkable, and the case was therefore considered non-syndromic. This step is crucial, as early detection of syndromic conditions such as familial adenomatous polyposis can have significant systemic implications [15].

Diagnostic challenges

Diagnosis of supplemental incisors is often complicated by their close resemblance to natural teeth. In our patient, careful intraoral examination, flossing tests, and radiographic evaluation (OPG and cephalogram) were essential to confirm the presence of distinct crowns and roots. Importantly, the left supplemental incisor exhibited crown attrition and root resorption, a finding with clinical implications for long-term prognosis. Although cone-beam computed tomography (CBCT) could have provided additional three-dimensional detail, conventional radiographs were sufficient for diagnosis and treatment planning in this case. Nevertheless, CBCT remains the gold standard, where root resorption or ectopic positions are suspected [16].

Treatment rationale

Management of supplemental incisors is highly individualized and depends on tooth morphology and position, associated pathology, and orthodontic considerations. Options include monitoring, extraction, and transplantation or orthodontic alignment of the supernumerary tooth. In Robertson et al.’s classic report, a supplemental tooth was transplanted across the arch to replace a compromised natural incisor. By contrast, our patient’s natural lateral incisors were healthy, while the left supplemental tooth displayed resorption and attrition. Additionally, generalized anterior spacing, increased proclination, and excessive incisor display necessitated space closure and incisor retraction for improved esthetics and function. Extraction of both supplemental teeth followed by fixed mechanotherapy was therefore the most appropriate choice, providing a predictable outcome and minimizing long-term risks. Alternative approaches, such as transplantation, were deemed unsuitable due to complete root development and unfavorable prognosis for autotransplant survival.

Comparison with literature

Contemporary case reports emphasize the rarity of bilateral supplemental laterals and highlight diverse treatment strategies. Yildirim and Bayrak reported early diagnosis in primary and permanent dentitions to prevent occlusal disturbances [17]. More recent reports describe both extraction and orthodontic alignment approaches depending on occlusion and esthetics [18,19]. Our case adds to this body of evidence by demonstrating the rationale for extraction in the presence of root resorption, proclination, and spacing, features that distinguish it from both historical cases [20] and recent reports where preservation was prioritized.

Limitations and future directions

The principal limitation of this report is that active orthodontic treatment is ongoing. While the rationale for extraction and treatment planning is presented, the absence of final alignment and long-term follow-up reduces the immediate clinical applicability of the report. We acknowledge that definitive outcomes, including periodontal health, stability, and esthetic results, are the most valuable component of any case report. A follow-up publication is planned to present treatment completion and long-term stability.

## Conclusions

This case underscores the importance of identifying and addressing rare dental anomalies such as bilateral supplemental maxillary lateral incisors. While these additional teeth may not cause immediate symptoms, they can still lead to functional or esthetic concerns if overlooked. Careful clinical and radiographic examination plays a key role in timely diagnosis, and tailoring the treatment plan to each patient ensures the best possible outcome. For clinicians, staying alert to such unusual presentations during routine check-ups is essential to providing thorough and effective care.
